# Predictors of willingness to accept pre-marital HIV testing and intention to sero-sort marital partners; risks and consequences: Findings from a population-based study in Cameroon

**DOI:** 10.1371/journal.pone.0208890

**Published:** 2018-12-19

**Authors:** Derick Akompab Akoku, Mbah Abena Tihnje, Elisabeth Oben Tarh, Elvis Enowbeyang Tarkang, Robinson Enow Mbu

**Affiliations:** 1 Community Research and Training Institute, Yaoundé, Cameroon; 2 Ministry of Public Health, Yaoundé, Cameroon; 3 School of Public Health, University of Health and Allied Sciences, Ho, Ghana; 4 Faculty of Medicine and Biomedical Sciences, University of Yaoundé 1, Yaoundé, Cameroon; The Chinese University of Hong Kong, HONG KONG

## Abstract

**Introduction:**

The objectives of this study was to investigate and compare levels of acceptability of pre-marital HIV testing; and intention to sero-sort future marital and its associated factors among unmarried adults in two cities in Cameroon.

**Methods:**

A population-based survey was conducted simultaneously in the cities of Kumba and Buea, located in the Southwest region of Cameroon. Data were collected from September to October 2016 by trained interviewers who administered questionnaires to eligible and consenting unmarried adults aged 21–35 years. Data were weighted and logistic regression analyses performed to identify significant predictors. The level of statistical significance was set at p< = 0.05.

**Results:**

A total of 1,406 respondents (767 in Kumba and 639 in Buea) participated in the study. In the pooled sample, the median age of respondents was 26 years (IQR = 23–29) and over half (54.8%) were males. Over 90% of respondents in both cities indicated their willingness to accept pre-marital HIV testing. Respondents who had previously tested for HIV in Kumba (AOR = 7.87; 95%CI, 4.02–15.44) were significantly more likely to accept premarital HIV testing than those who had never tested for HIV. In Kumba, older age (AOR = 0.42; 95%CI, 0.18–0.96) and those unemployed (AOR = 0.22; 95% CI, 0.06–0.76) were significantly less likely to accept pre-marital HIV testing. In Buea males (AOR = 0.64 95% CI, 0.45–0.89) who would test HIV negative would be significantly less likely to accept to marry an HIV positive partner. In Buea, respondents who indicated a moderate risk of contracting HIV (AOR = 1.71; 95%CI, 1.09–2.66, p = 0.018) were significantly more likely to accept to marry an HIV positive partner. The major limitation of the study was that a hypothetical situation was used to ask respondents about their willingness to accept pre-marital HIV testing rather than actual HIV test acceptance.

**Conclusions:**

Most respondents expressed their willingness to undergo pre-marital HIV testing. However, majority of respondents who would test HIV negative would refuse to marry their partner who tests HIV positive. These findings suggest that interventions to reduce HIV infection and fight against stigma and discrimination should be reinforced at community level.

## Introduction

Cameroon has a mixed HIV epidemic, with an estimated HIV prevalence of 4.3% among the general population [1]. However, the HIV prevalence is higher among certain subpopulations (36% among female sex workers and 37% among men who have sex with men) [2, 3]. The government through the Ministry of Health with support from national and international partners has allocated substantial resources to promote HIV testing due to evidence that early diagnosis and linkage to anti-retroviral therapy (ART) reduces transmission and improves health outcomes [4]. Despite recent efforts to increase HIV testing in the country, only an estimated 23.8% of adults aged 15–49 years know their HIV status[5] and as a result many individuals are unaware of their HIV status. Previous research conducted in other countries in sub-Saharan Africa (SSA) has also reported evidence of low HIV testing rates [6, 7].This undermines the goals of HIV prevention and treatment programs and further fuels HIV transmission in the community.

The low HIV testing rates in these countries has prompted calls for a more pragmatic strategy to reduce the spread of HIV. One strategy that has been adopted by a number of religious institutions and local governments is mandatory pre-marital HIV testing as a means to combat the spread of HIV among marital partners [8–11]. This is based on evidence that HIV sero-discordant couples in stable relationships are a major source of new infections [12, 13] and sero-discordancy has been estimated to occur in 8–40% of couples [14, 15]. A study conducted in Zambia and Rwanda showed that an estimated 50% of new heterosexual HIV infections occurred among sero-discordant couples [12]. Recent studies also show that in HIV sero-discordant couples, 65–85% of new infections are acquired from the married/cohabiting partner [16]. HIV negative individuals in sero-discordant marriages have a higher risk of being infected by their HIV positive partners [17].

Pre-marital HIV testing is a highly contentious issue and there are arguments whether it should be mandatory or voluntary [13, 18]. Some posit that making it mandatory is unethical as it infringes the human rights of individuals and increases stigmatization to those who test HIV positive [19–21]. A number of proponents, including women’s groups believe that mandatory pre-marital HIV testing and denying discordant marriages will protect women from HIV infection upon marriage [9]. In Malaysia, major stakeholders viewed mandatory pre-marital HIV testing as an effective HIV prevention policy and was considered as a positive step towards HIV prevention in the community [10]. Nevertheless, mandatory pre-marital HIV testing is against the World Health Organizations’ (WHO) recommendation which discourages all non-voluntary forms of HIV testing [22].

Despite this controversy, and the lack of scientific evidence on the preventive benefits of pre-marital HIV testing, some religious groups in SSA have made pre-marital HIV testing mandatory as a pre-condition for marriage in church with a view of denying marriage in the case where there is sero-discordancy [9]. Some religious leaders are of the opinion that mandatory HIV testing is a way of protecting those who are HIV negative from being infected [10, 23]. A study conducted in Nigeria among 317 members of a religious group referred for pre-marital HIV testing found that 7.8% individuals tested positive [18]. Another study conducted among 571 unmarried youths in Ibadan, Nigeria found that 82.8% believed that mandatory pre-marital HIV testing could reduce the spread of HIV. However, 43.8% indicated that it will increase stigma for those who test HIV positive [24]. A study in Kenya demonstrated that young women were motivated to get tested for HIV because of their intention to get married in the future [25] which may suggest that they considered marriage important in their lives to have a family and bear children.

There is a growing body of literature on the phenomenon of sero-sorting. Sero-sorting has been defined as a practice where an individual intentionally selects his/her sexual partner (in this case a spouse) known to be of the same HIV sero-status, to reduce the risk of acquiring or transmitting HIV after having unprotected sex [26, 27]. Some authors have suggested that sero-sorting reduces the risk of acquiring or transmitting HIV as partners share the same HIV status [26, 28]. It is widely acknowledged that for sero-sorting to be effective, both partners need to know the HIV status of each other and pre-marital HIV testing offers an opportunity where prospective couples can know their HIV status before marriage. To date, most research on sero-sorting has been conducted among most-at risk population groups such as men who have sex with men [28–30] with limited research among the general population.

As in other African countries [9, 31] some Pentecostal churches in Cameroon require HIV testing as a pre-condition for marriage in church. In spite the controversial nature of mandatory pre-marital HIV testing, there is no data at population level on public attitudes towards pre-marital HIV testing and little is known whether young unmarried adults would accept this HIV testing approach. Furthermore, no research has investigated intentions to sero-sort marital partners in the country. The objectives of this study were to (1) determine and compare the level of willingness to accept pre-marital HIV testing and its associated factors, and (2) investigate intention to sero-sort future marital partners among unmarried adults (aged 21–35 years) in two urban cities in Cameroon. The findings of this study will fill the gaps in literature and contribute to better inform HIV prevention strategies and interventions in the country.

## Materials and methods

### Study design and setting

The study was a population-based study conducted in two cities (Kumba and Buea) located in the Southwest region of Cameroon. The Southwest region is one of the two English-speaking regions of Cameroon. Kumba and Buea were selected for this study because they are the two principal cities in the region (Kumba is the economic capital while Buea is the administrative capital of the Southwest region). The Southwest region is made up of six administrative divisions (Fako, Meme, Ndian, Kupe-Manengouba, Lebialem and Manyu) and has an estimated HIV prevalence of 5.7%[1]. Kumba is also the administrative headquarters of Meme division and has a population of about 191,584 [32]. It is divided into three districts (Kumba 1, Kumba 2 and Kumba 3). The inhabitants come from a wide variety of cultural backgrounds and socio-economic groups. Most of them are involved in the agricultural and retail sectors of the economy. Buea is located about 74 km from Kumba. It has an estimated population of 151,263 inhabitants [32]. It is located on the eastern slopes of Mount Cameroon (4100m above sea level, the second highest mountain in Africa after the Kilimanjaro Mountain). Buea is made up of an ethnically and culturally diverse population. Agriculture has been the main activity among most residents although many individuals are involved in the retail industry. In recent years, the city has become a centre of learning with many academic institutions.

### Study population and sampling

This population-based survey was conducted among adults aged 21–35 years resident in the cities of Kumba and Buea. The minimum sample size for each city was derived from the formula: n = [z^2^ x p (1-p)] DEFF / [m^2^]; where n = the minimum required sample size for the study; z = the standard deviation for a 2-tailed test at 95% confidence level (1.96); p (50%) = the estimated population parametre (given that there was no previous data on intention to sero-sort future marital partners in SSA); DEFF (2.0) the design effect, m = the margin of error (6%) and a non-response rate of 15%. The minimum estimated sample size for each city prior to data collection was 628 individuals. Respondents were selected through a modified version of multi-stage cluster sampling technique. At the first stage, we used census data from the National Institute of Statistics and data from the administrative office and selected 24 neighbourhoods (clusters) in Kumba and 20 neighbourhoods (clusters) in Buea based on probability proportionate to size. The secondary sampling units were every third household in the selected neighbourhoods; and the tertiary sampling units were all eligible respondents in selected households. All unmarried permanent residents in the selected households or visitors who spent the previous night, who were aged between 21–35 years and willing to provide written informed consent were eligible for the study. Those who lacked the cognitive and intellectual capacity as well as refused to provide written informed consent were excluded from the study.

### Data collection

Data were collected from September to October 2016 simultaneously in Kumba and Buea at household level. In the sampled neighbourhoods (clusters), trained interviewers selected a random starting direction by spinning a pen and choosing the direction. Interviewers randomly visited every third household (along this line until the boundary of that neighbourhood was reached), screened and identified eligible respondents. Data were collected from every third household because neighbouring households tend to be more similar to each other [33]. All eligible respondents were explained the objectives of the survey, their rights to participate as well as to opt-out at any time. They were informed that their responses would be kept confidential and there would be no way to identify them after the interview as no identifiable information was collected. Only eligible respondents who were present in the household at the time of the visit, who expressed interest and provided written informed consent were invited to participate. Data were collected using a pre-tested and validated paper-based questionnaire (S1 Questionnaire) in English. Data were not collected from non-responders. Those who refused to participate indicated lack of time and interest in the study.

### Measures

#### Dependent variables

This study examined two main outcome variables. The first outcome variable was “willingness to accept a pre-marital HIV test”. This variable was assessed by asking respondents: “Would you be willing to have an HIV test before marriage, if requested?” The response option was “No” or “Yes”. To assess barriers to testing, those who responded “No” were asked: “Why would you refuse to have an HIV test a few weeks before your marriage?”

The second outcome variable was “acceptance to marry an HIV positive partner” after a sero-discordant test. Respondents were explained the meaning of sero-discordancy and asked: “If the results of a pre-marital HIV test show that you are HIV negative while your partner is HIV positive, would you still marry your HIV positive partner?” The response option was “No” or “Yes”. All respondents were further asked: “How would you feel if the results show that you were HIV negative while your partner is HIV positive?” Respondents were further asked if they will seek support to help them manage the situation.

#### Independent variables

The independent variables included sociodemographic variables: age was classified in three age groups (21–25, 26–30 and 31–35 years); gender (male or female), educational attainment-in terms of highest level of education completed (primary, secondary, high school, and university); employment status (student, unemployed, and employed), and religious affiliation (Catholic, Presbyterian, Pentecostal, and Others). Other independent variables included: “currently in a sexual relationship”, “knowledge of current sexual partner’s HIV status”, “knowledge of someone currently living with HIV”, “knowledge of someone who has died of AIDS” and “self-perceived risk of contracting HIV”. We measured self-perceived risk of contracting HIV by asking respondents the following question: “How do you rate your personal risk of contracting HIV?” Perceived risk of contracting HIV was coded “1” if respondents had considered themselves to have no risk, “2” if they considered themselves to have small risk, “3” if they considered themselves to have moderate risk, and “4” if they considered themselves to have high risk.

### Ethical considerations

The study protocol was reviewed and approved by the Cameroon National Research Ethical Committee for Human Health (No: 2016/11/841/CE/CNERSH/SP). Administrative authorisation was obtained from the Southwest Regional Delegation for Public Health. All respondents were explained the objectives of the study and those who expressed willingness to participate provided written informed consent. Respondents were assured that data collected will be kept strictly confidential.

### Statistical analysis

Data analyses were performed using STATA 13.0 (StataCorp, College Station, TX). The survey data (S1 Study Dataset) were weighted to account for unequal probabilities of selection of neigbourhoods and respondents based on the two-stage sampling design. This was performed in STATA using the “**svyset**” command taking into account relevant survey design parameters including the sampling weights and finite population corrections[34]. To adjust for standard errors due to the clustered nature of the data (because respondents were recruited from different neighborhoods), all analyses were performed using the STATA “**svy**” prefix command. Descriptive analyses such as frequencies and percentages, median and interquartile range (IQR) were used to identify participants’ characteristics. The Chi square (χ2) test was performed to determine any significant differences between the characteristics of respondents in Kumba and Buea.

Univariate logistic regression analyses were performed to identify co-variates associated with the outcome variables. For the outcome variable: “willingness to accept pre-marital HIV test”, all the “No” responses where coded as “0” and the “Yes” responses coded as “1”. Similarly, for the outcome variable: “acceptance to marry an HIV positive partner”, all the “No” responses were coded as “0” and the “Yes” responses coded as “1”. Only variables with a p-value <0.25 in univariate analysis (S1 and S2 Tables) were entered into multivariate logistic regression models to examine the independent predictors of the dependent variables. Due to collinearity, the variable “currently in a sexual relationship” was eliminated from the model because it was correlated with "knowledge of sexual partners HIV status". Unadjusted odds ratio (ORs) and adjusted odds ratios (AORs) as well as 95% confidence intervals (CI) were reported. In multivariate models, controlling for potential confounders, we considered a two-sided p-value ≤0.05 to determine statistical significance. This paper has been prepared in conformity with the STROBE guidelines, recommended by the Enhancing the Quality and Transparency of Health Research (EQUATOR) network (S1 STROBE Checklist).

## Results

### Demographic and health characteristics of respondents

**Table 1** shows the weighted results of respondents’ characteristics at an aggregate level and by city. A total of 1,756 respondents (970 in Kumba and 789 in Buea) met survey eligibility criteria after screening. Of these, 1,406 (767 in Kumba and 639 in Buea) respondents agreed to participate and were successfully interviewed (response rate: pooled sample [80.0%]; Kumba [79%] and Buea [81%]). There was no significant difference in survey response rate between both cities. In the pooled sample, the median age of respondents was 26 years (IQR = 23–29) and 46.2% were aged 21–25 years. Over half (54.8%) were males; there were more male respondents in Kumba compared to Buea (58.1% vs 50.9%, p = 0.003). About 35.8% of respondents in the pooled sample attained high school education; respondents in Buea were significantly more likely to have attained high school education compared to those in Kumba (38.3% vs 33.7%,p = 0.015). More than half (62.7%) were employed; respondents in Kumba were significantly more likely to be employed than those in Buea (64.1% vs 61.0%, p = 0.022).

**Table 1:** Demographic and health characteristics of respondents.

Characteristics	Pooled sample (N = 1,406)	Kumba (N = 767)	Buea (N = 639)	P-value
	n (%)	n (%)	n (%)	
**Age group(years)**				0.430
21–25	650 (46.2)	349 (45.5)	301 (47.1)	
26–30	507 (36.1)	287 (37.4)	220 (34.4)	
31–35	249 (17.7)	131 (17.1)	118 (18.5)	
**Gender**				0.003
Female	635 (45.2)	321 (41.9)	314 (49.1)	
Male	771 (54.8)	446 (58.1)	325 (50.9)	
**Educational attainment**				0.015
Primary school	352 (25.0)	208 (27.1)	144 (22.5)	
Secondary school	318 (22.6)	185 (24.1)	133 (20.8)	
High school	503 (35.8)	258 (33.7)	245 (38.3)	
University	233 (16.6)	116 (15.1)	117 (18.3)	
**Employment status**				0.022
Student	293 (20.3)	141 (18.4)	152 (23.8)	
Unemployed	231 (16.4)	134 (17.5)	97 (15.2)	
Employed^1^	882 (62.7)	492 (64.1)	390 (61.0)	
**Religion**				0.209
Catholic	368 (26.2)	192 (25.0)	176 (27.5)	
Presbyterian	361 (25.7)	208 (27.1)	153 (23.9)	
Pentecostal	363 (25.8)	205 (26.7)	158 (24.7)	
Others^2^	314 (22.3)	162 (21.1)	152 (23.8)	
**Currently in a sexual relationship**				0.205
No	418 (29.7)	238 (31.0)	180 (28.2)	
Yes	988 (70.3)	529 (67.0)	459 (71.8)	
**Know current sexual partner’s HIV status**^**3**^				<0.001
No	335 (33.9)	213 (40.3)	122 (26.6)	
Yes	653 (66.1)	316 (59.7)	337 (73.4)	
**Know someone living with HIV**				<0.001
No	956 (68.0)	567 (73.9)	389 (60.9)	
Yes	450 (32.0)	200 (26.1)	250 (39.1)	
**Know someone who has died of AIDS**				0.001
No	763 (54.3)	446 (58.2)	317 (49.6)	
Yes	643 (45.7)	321 (41.8)	322 (50.4)	
**Self-perceived risk of contracting HIV**				<0.001
No risk	332 (23.6)	178 (23.2)	154 (24.1)	
Small risk	479 (34.1)	236 (30.8)	243 (38.0)	
Moderate risk	346 (24.6)	188 (24.5)	158 (24.7)	
High risk	249 (17.7)	165 (21.5)	84 (13.2)	
**Previously tested for HIV**				0.153
No	437 (31.1)	227 (29.6)	210 (32.9)	
Yes	969 (68.9)	540 (70.4)	429 (67.1)	

Notes

Numbers are unweighted, percentages are weighted; p- values were calculated from Chi-square tests

^1^Employed: Part-time, Full time or self-employed

^2^Other Religion included: Baptist, Islam, Apostolic, Jehovah’s Witness etc

^3^ Only for those who were currently in a sexual relationship

About 70.3% (988/1,406) respondents in the pooled sample were in a sexual relationship at the time of the study (Table 1). Among these, 66.1% knew the HIV status of their current sexual partner. Respondents in Buea were significantly more likely to have known the HIV status of their current sexual partner (73.4% vs 59.7%, p<0.001) compared to those in Kumba. About 68.0% (956/1,406) of respondents did not know anyone living with HIV and 54.3% did not know someone who had died of AIDS. However, respondents in Buea were significantly more likely to have known someone who was living with HIV (39.1% vs 36.1%, p<0.001) and someone who had died of AIDS (50.4% vs 41.8%, p = 0.001) compared to those in Kumba. In both cities, 34.1% of respondents indicated that they had a “small risk” of contracting HIV. Respondents in Buea were significantly more likely to have perceived a “small risk” of contracting HIV than those in Kumba (38.0% vs 30.82%, p<0.001).

### Factors associated with willingness to accept pre-marital HIV testing

Most of the respondents in Kumba (94.5%) and Buea (95.5%) indicated their willingness to accept a pre-marital HIV test if requested. Those who indicated that they will refuse to accept a pre-marital HIV test cited; fear of stigma (3.3%); discrimination and rejection (2.2%), lack of confidentiality (1.64%), cost (0.36%) and fear of death (0.21%) as barriers to HIV testing before marriage.

Table 2 shows results of weighted multivariate logistic regression analysis of factors associated with willingness to accept pre-marital HIV testing. Our analyses showed that in Kumba, older age (AOR = 0.42; 95%CI, 0.18–0.96, p = 0.04) and those unemployed (AOR = 0.22; 95% CI, 0.06–0.76, p = 0.018) were significantly less likely to accept pre-marital HIV testing. However, respondents in Kumba who had previously tested for HIV (AOR = 7.87; 95%CI, 4.02–15.44, p<0.001) were significantly more likely to accept pre-marital HIV testing than those who had never tested for HIV. In Buea, males (AOR = 0.38; 95%CI, 0.15–0.98, p = 0.047) were significantly less likely to accept pre-marital HIV testing than females.

**Table 2:** Multivariate logistic regression analysis of factors associated with “willingness to accept pre-marital HIV testing”.

Independent variables^1^	Kumba		Buea	
	AOR (95% CI)	P-value	AOR (95% CI)	P-value
**Age group (years)**				
21–25	1.0		1.0	
26–30	0.57 (0.28–1.15)	0.118	0.58 (0.18–1.87)	0.363
31–35	0.42 (0.18–0.96)	0.040	0.35 (0.11–1.34)	0.081
**Gender**				
Female	1.0		1.0	
Male	0.67 (0.33–1.36)	0.269	0.38 (0.15–0.98)	0.047
**Employment status**				
Student	1.0			
Unemployed	0.22 (0.06–0.76)	0.018		
Employed^2^	0.33 (0.10–1.08)	0.069		
**Religion**				
Catholic	1.0			
Presbyterian	0.96 (0.38–2.43)	0.934		
Pentecostal	1.01 (0.40–2.56)	0.978		
Others^3^	0.44 (0.19–1.03)	0.060		
**Know current sexual partner’s HIV status**				
No			1.0	
Yes			2.30 (0.83–6.31)	0.105
**Previously tested for HIV**				
No	1.0		1.0	
Yes	7.87 (4.02–15.44)	<0.001	1.53 (0.61–3.88)	0.360

Notes

^1^ Variables that were not significant in univariate analysis (i.e., p-value<0.25) in Kumba, and Buea were excluded from the table. In Kumba, the variables age group, gender, employment status, religion and previously tested for HIV were entered in the multivariate model. In Buea, the variables age group, gender, know current sexual partners HIV status and previously tested for HIV were entered in the multivariate model.

^2^Employed: Part-time, Full time or self-employed

^3^Other religion included: Baptist, Islam, Apostolic, Jehovah’s Witness etc.

### Emotional reactions following a sero-discordant HIV test result

**Fig 1:**
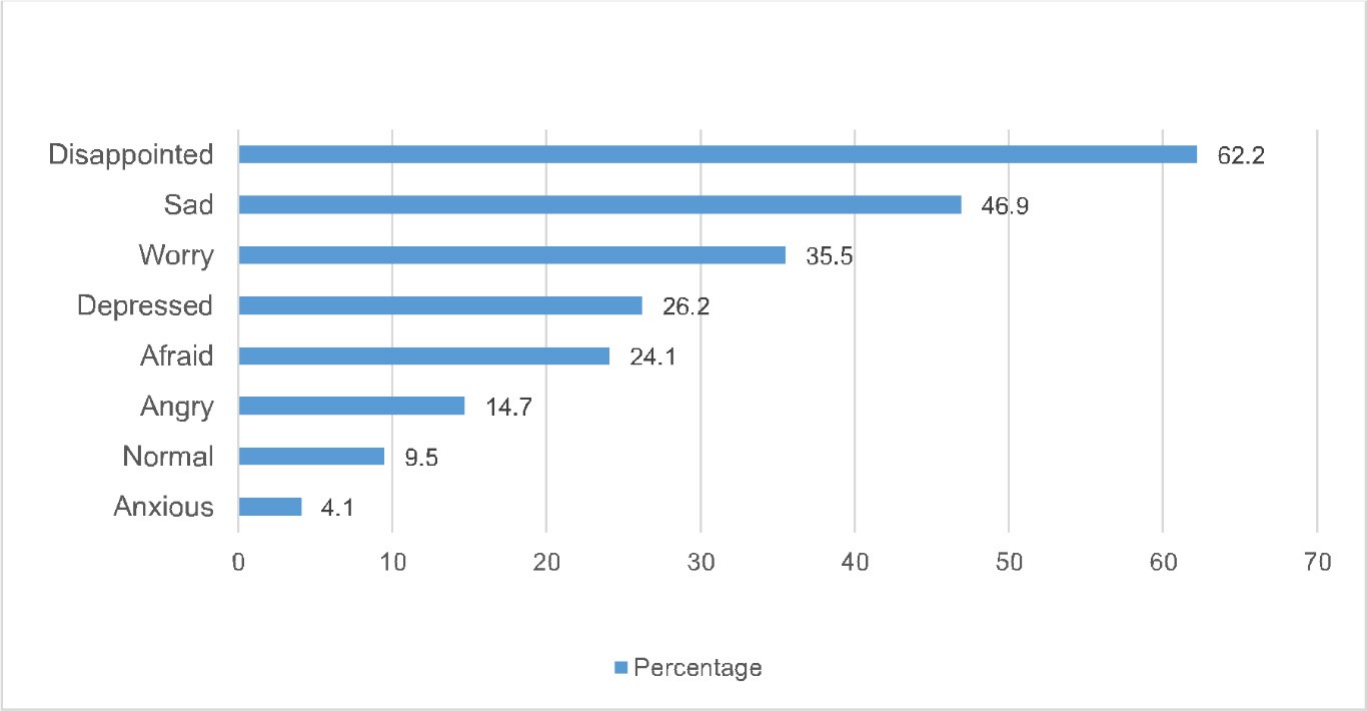
Respondents’ emotional responses in the event of a sero-discordant pre-marital HIV test result (n = 1,406). **Note:** Percentages are weighted and the total may sum up to more than 100% as multiple responses were permissible.

**Fig 1** shows respondents’ emotional/psychological reactions in the event of a sero-discordant pre-marital HIV test result. Respondents were asked, “How would you feel if a pre-marital HIV test shows that you are HIV negative while your partner is HIV positive?” In the pooled sample, the most frequently cited emotional reactions were disappointed (62.2%), sad (46.9%) and worry (35.5%).

### Seeking support following a sero-discordant pre-marital HIV test results

**Fig 2:**
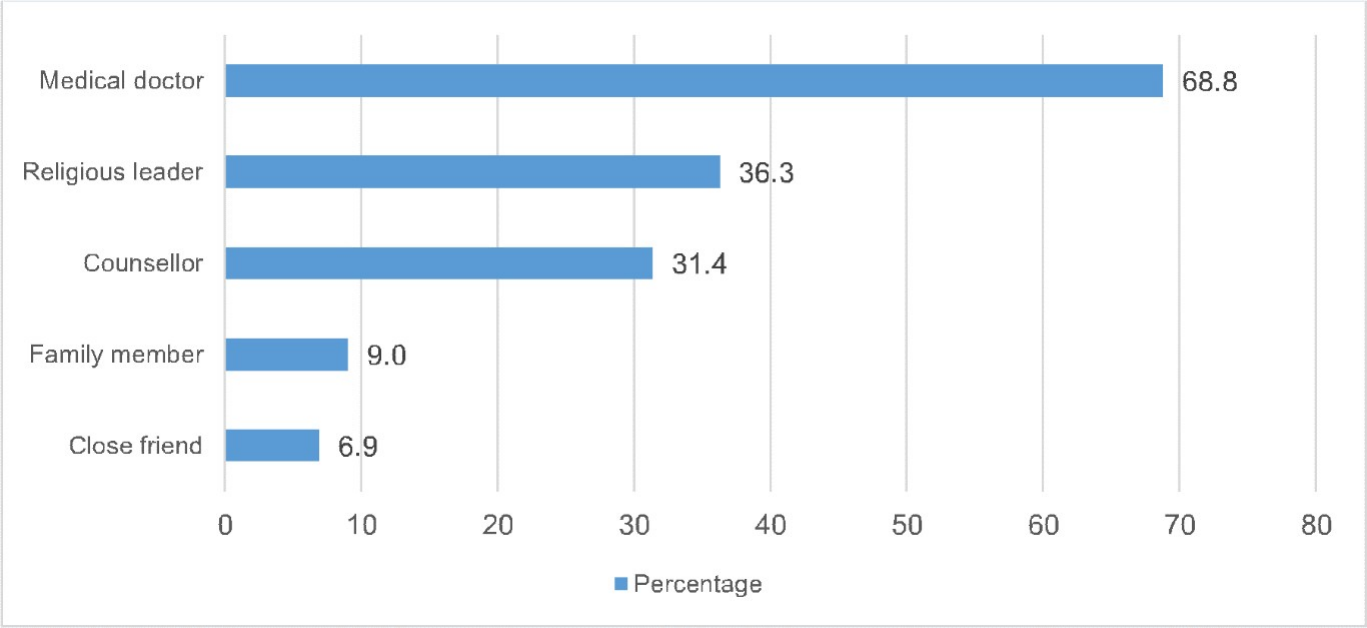
Person from whom respondents will seek support (n = 1,406). **Note:** Percentages are weighted and the total may sum up to more than 100% as multiple responses were permissible.

**Fig 2** shows the person from whom respondents would seek support following a sero-discordant pre-marital HIV test result with their partner (i.e., in a situation where the respondent tests HIV negative while their partner tests HIV positive). In the pooled sample, 83.6% indicated that they will seek support from someone (84.2% in Kumba versus 82.8% in Buea). Overall, the most frequently cited individual from whom they will seek support was a medical doctor (68.8%), followed by a religious leader (36.3%). In Kumba, males (AOR = 0.62, 95%CI, 0.42–0.92, p = 0.019) and those who attended high school (AOR = 0.55, 95% CI, 0.33–0.92, p = 0.024) were less likely to seek support from someone (data not shown). In Buea, we did not find any significant relationship between any of the independent variables and seeking support from someone after a pre-marital sero-discordant HIV test result.

### Acceptability to marrying an HIV positive partner

**Fig 3:**
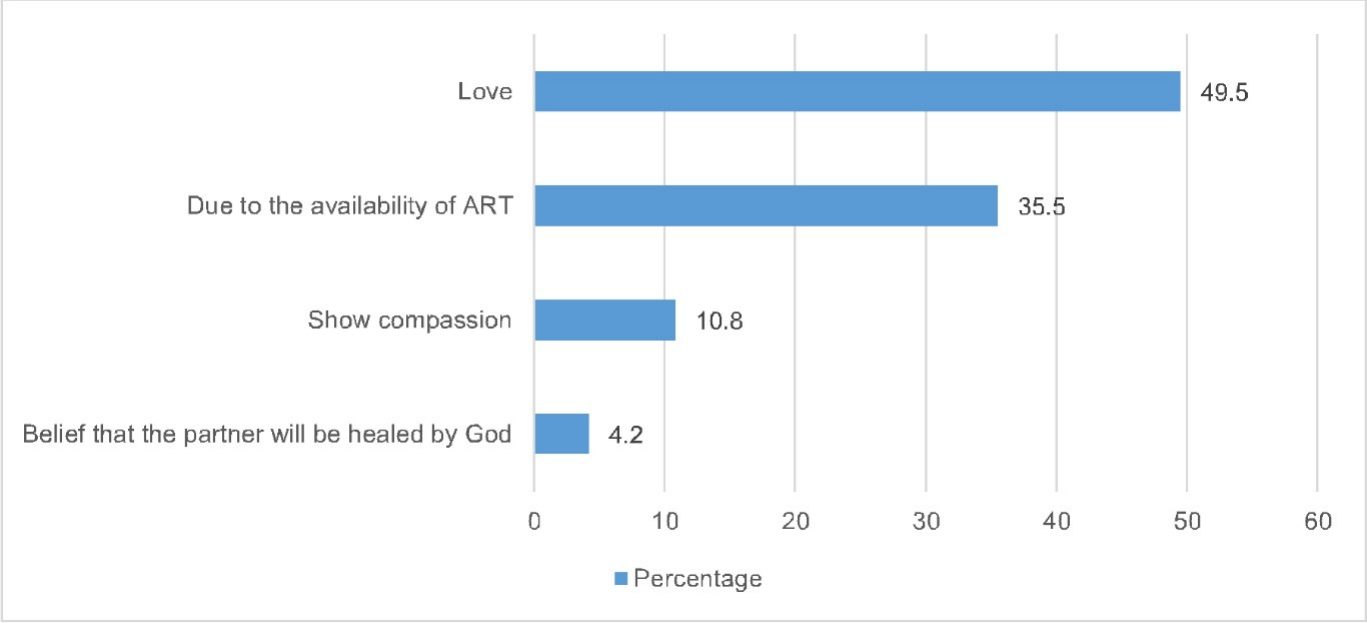
Reasons for marrying an HIV positive partner (n = 380)^1^. **Note:** ^1^A total of 380 individuals indicated that they will marry an HIV positive spouse. Percentages are weighted, and the total may sum up to more than 100% as multiple responses were permissible.

Most of the respondents in Kumba (73.7%) and Buea (72.1%) indicated that if they test HIV negative while their partner tests HIV positive, they will refuse to marry the partner. There was no statistical significant difference in refusal rates between both cities. Among those who indicated that they will still marry their HIV positive partner (26.3% in Kumba versus 27.9% in Buea), the main reasons provided was because of love (49.5%) and the availability of ART (35.5%) for HIV treatment (**Fig 3**).

### Factors associated with “acceptability to marry an HIV positive partner”

**Table 3** shows results of weighted multivariate logistic regression analysis for acceptance to marry an HIV positive partner after a pre-marital HIV test. Although not a significant predictor, we found that Pentecostal Christians in Kumba (AOR = 1.51; 95% CI, 0.91–2.49) were more likely than respondents in other religious groups to accept to marry an HIV positive partner. In Buea, male gender (AOR = 0.64; 95%CI, 0.45–0.89, p = 0.009) and Pentecostal Christians (AOR = 0.62; 95% CI, 0.39–0.98, p = 0.045) were significantly less likely to accept to marry a partner who tests HIV positive. Furthermore, respondents in Buea who indicated a moderate risk of contracting HIV (AOR = 1.71; 95%CI, 1.09–2.66, p = 0.018) were significantly more likely to accept to marry an HIV positive partner.

**Table 3:** Multivariate logistic regression analysis of factors associated with “acceptance to marry an HIV positive partner”.

Independent variables^1^	Kumba		Buea	
	AOR (95%CI)	P-value	AOR (95%CI)	P-value
**Age group (years)**				
21–25	1.0			
26–30	0.79 (0.51–1.23)	0.306		
31–35	1.44 (0.87–2.41)	0.156		
**Gender**				
Female	1.0		1.0	
Male	0.83 (0.57–1.21)	0.339	0.63 (0.45–0.89)	0.009
**Employment status**				
Student	1.0			
Unemployed	0.96 (0.49–1.88)	0.920		
Employed^2^	1.21 (0.71–2.09)	0.476		
**Religion**				
Catholic	1.0		1.0	
Presbyterian	1.00 (0.60–1.68)	0.984	0.70 (0.45–1.10)	0.124
Pentecostal	1.51 (0.91–2.49)	0.112	0.62(0.39–0.98)	0.045
Others^3^	1.17 (0.68–2.00)	0.563	0.66(0.42–1.04)	0.074
**Know current sexual partner’s HIV status**				
No	1.0			
Yes	1.23 (0.84–1.81)	0.288		
**Know someone living with HIV**				
No	1.0			
Yes	1.15 (0.75–1.74)	0.509		
**Self-perceived risk of contracting HIV**				
No risk			1.0	
Small risk			0.72(0.46–1.10)	0.130
Moderate risk			1.71(1.09–2.66)	0.018
High risk			0.91(0.52–1.61)	0.761

Notes

^1^ Variables that were not significant in univariate analysis (i.e., p-value<0.25) in Kumba, and Buea were excluded from the table. In Kumba, the variables; age group, gender, employment status, religion, know current sexual partners HIV status and “know someone living with HIV” were entered in the multivariate model. In Buea, the variables; gender, religion and perceived risk of contracting HIV were entered in the multivariate model.

^2^Employed: Part-time, Full time or self-employed

^3^Other religion included: Baptist, Islam, Apostolic, Jehovah’s Witness etc.

## Discussion

This study aimed to examine the willingness to accept pre-marital HIV testing and intention to sero-sort future marital partners among unmarried adults in two urban cities in Cameroon. Our findings demonstrate that a majority (>90% in both cities) of respondents indicated their willingness to accept a pre-marital HIV test. However, most individuals who would test HIV negative would less likely accept to marry their partner who tests HIV positive. In Buea, males were less likely to accept pre-marital HIV testing than females. This finding is consistent with those of previous studies that have reported that HIV testing rates among men are low because of their unwillingness to get tested [35–37].

In Kumba, those who were unemployed were less likely to accept pre-marital HIV testing. Their refusal could be due to some of the barriers identified in this study including the lack of confidentiality of HIV test results; and the fear of stigma and discrimination associated with pre-marital HIV testing[9]. Additionally, their fear of marriage denial in the event of a discordant test may lead their peers and community to conclude that one or both partners is HIV positive, and secrecy may no longer be possible to maintain under such circumstances[9]. In Kumba and Buea, we found that respondents who attend Pentecostal churches were more likely to accept pre-marital HIV testing. This finding could be because it is mostly Pentecostal churches that require HIV testing for their church members as a pre-condition for marriage in church [9]. As these respondents are Christians of these churches, they have a higher likelihood of respecting the requirements of the church.

A positive HIV test is usually associated with negative emotional reactions and this can be severe for intending couples to learn of a sero-discordant pre-marital HIV test result. The emotions/psychological reactions in this case will affect both partners. Our finding supports those of previous studies (although not in the context of pre-marital HIV testing) which show that the receipt of a positive HIV test result was associated with negative emotional reactions including sadness and depression [38, 39]. For the partner diagnosed HIV positive, this could be a stressful period and coping mechanisms are required. In a study conducted to examine illness appraisals among 100 newly diagnosed HIV positive individuals, researchers found that strong negative emotions of sadness, fear, upset, and anger accompanied the participants’ appraisal and the thought of HIV meant they would die[40].

The most likely individuals mentioned for support were a medical doctor and a religious leader. This finding demonstrates the important role and trust that respondents have with medical practitioners and their belief that they are best placed to provide medical advice and risk reduction counselling. Studies have reported that religion and spirituality plays an essential role in mitigating the emotional reactions following a positive HIV test result [41, 42]. Among HIV positive individuals, higher levels of religion/spirituality have been associated with improvements in their psychological, mental and physical health outcomes [43]. In this study, some respondents (Fig 3) indicated that they will still marry their HIV positive partners because they believe that the HIV positive partner will be healed by God. For example, in Kumba, Pentecostal Christians who would test HIV negative would be more likely to accept to marry an HIV positive partner. It is plausible that these Pentecostal Christians may have felt that through prolonged prayers their partners would be healed by God. In many parts of Africa, HIV positive individuals usually seek healing from faith healers [44] and this explains why some respondents mentioned seeking support from religious leaders.

The majority of the respondents indicated that if a pre-marital HIV test results reveal that they were HIV negative while their partner was HIV positive (a sero-discordant test), they will refuse to marry their HIV positive partner. This finding demonstrates the phenomenon of sero-sorting (as HIV negative individuals will prefer to marry a partner who is also HIV negative) when choosing marital partners. Our finding is somewhat similar to that of a study conducted in Uganda, were 79% of participants who intended to get married indicated that the HIV status of their partners would be a key consideration when choosing their partners. Among those whom the HIV status was a key consideration, 50% preferred to have a sero-negative spouse [45].

In Buea, respondents who indicated a moderate risk of contracting HIV were more likely to accept to marry a partner who tests HIV positive after a pre-marital HIV test. Although more evidence is required, this finding may suggest that those who would test HIV positive would prefer to marry a partner who also tests HIV positive, a finding which supports the phenomenon of sero-sorting among HIV positive individuals. In Buea, we found that women were more likely to accept to marry their HIV positive partner. A plausible explanation for this could be due to women’s weaker position in negotiating sexual relationship, their economic vulnerability and dependence on men for survival [46]. It is important to keep in mind that even if HIV negative partners enter into marriage, there is a risk that if one partner engages in extra-marital unprotected sexual partnerships, this increases their risk of contracting HIV with a potential of transmitting the virus to their spouse[47, 48].

For some of the few respondents who indicated that they will still marry their HIV positive spouse, the availability of ART was indicated as one of the reasons. There is scientific evidence that among heterosexual sero-discordant couples if the HIV positive partner is on sustained ART (and achieves viral suppression) in addition to counselling and condom use, there is about 96% reduction in the risk of HIV transmission to the un-infected partner [4]. Another study conducted among 3,400 HIV-1 sero-discordant heterosexual couples from seven African countries, showed that the use of ART was associated with a 92% reduction in the risk of HIV transmission to their HIV negative partners [49].

Although it is possible for an HIV negative individual to marry an HIV positive partner and give birth to HIV negative children [50], there is growing concern of marital instability among sero-discordant couples [51]. It has been reported that the rate of marriage dissolution and divorce among sero-discordant couples is high. For example, in a prospective study among HIV sero-discordant couples in Kenya, the incidence of relationship dissolution was high with 24% of couples reporting separation during 1–2 years of follow-up. Over half (53%) of participants who reported separation indicated that it was caused by sero-discordancy [52]. In Kumba and Buea, 26.3% and 27.0% of respondents respectively indicated that they will still marry their partner even if their partner tested HIV positive. However, there is evidence that marriages in which only the wife is HIV-positive are at an elevated risk of divorce compared to concordant negative unions [52, 53].

The strength of our study relates to the fact we used a robust sampling technique to select a representative sample of respondents in both cities. The sample size was adequate and the findings could be generalized to the entire study population in both cities. Additionally, appropriate statistical techniques were used to adjust for clustering effect on the variances of point estimates. The usefulness of these findings should be interpreted bearing in mind the following limitations. First, we used a hypothetical situation (willingness to receive pre-marital HIV testing) rather than actual HIV test acceptance. The hypothetical nature of the question may have affected our data. Second, because of the sensitive nature of some questions and the fact that data were collected via interviews it may be subject to social desirability bias. Third, due to the cross-sectional nature of the study, the cause-effect relationship cannot be inferred. Fourth, as this was a household survey, we excluded individuals in non-household settings (e.g., markets, prisons, schools and government offices). Finally, it is likely that both response bias and non-response bias (non-responders and responders may have differed in some of the parameters assessed) may have affected our data. Unfortunately, data were not collected from non-responders, so it was difficult to establish if there were significant differences between both groups.

Despite these limitations, this study has enable us understand levels of acceptability of pre-marital HIV testing, its potential risks and consequences. Further studies (both qualitative and quantitative) are required to investigate how individuals conceptualise sero-sorting and to further examine public attitudes towards pre-marital HIV testing. Additional studies are needed to explore the phenomenon of sero-sorting among HIV positive individuals.

## Conclusions

Our findings show that most unmarried young adults would be willing to accept pre-marital HIV testing and they intend to sero-sort their future marital partners. This study shows that premarital HIV testing is associated with risks and negative social and health consequences. If marriage is denied in the event of sero-discordancy among prospective couples it will become difficult to maintain confidentiality and this will negatively affect the emotional and psychological wellbeing of prospective couples. Our findings suggest that local authorities and religious leaders need to consider these associated risks prior to enacting regulations on pre-marital HIV testing. Prospective couples should be counselled on the benefits of knowing their HIV status before marriage and given the opportunity to decide whether to undergo HIV testing before marriage. Overall, our study findings also underscore the need for relevant stakeholders including parents, teachers, religious leaders and health professionals to reinforce HIV prevention messages and interventions targeted at adolescents and young adults that will enable them protect themselves from HIV infection.

## Supporting information

S1 STROBE Checklist Checklist of items that should be included in reports of cross-sectional studies. (DOC)

S1 Questionnaire This is the questionnaire that was used to collect data for this study. (DOCX)

S1 Study Dataset This is the study dataset. (XLS)

S1 Table Univariate logistic regression analysis of factors associated with willingness to accept pre-marital HIV testing. (DOCX)

S2 Table Univariate logistic regression analysis of factors associated with acceptance to marry an HIV positive partner. (DOCX)
