# Supplementary material for: Predictors of willingness to accept pre-marital HIV testing and intention to sero-sort marital partners; risks and consequences: Findings from a population-based study in Cameroon
Source: PLoS One. 2018 Dec 19;13(12):e0208890. doi: 10.1371/journal.pone.0208890 (PMC6300297; doi:10.1371/journal.pone.0208890)
Supplement: S1 STROBE Checklist — (DOC) [file pone.0208890.s001.doc]

S1. STROBE Checklist—Checklist of items that should be included in reports of cross-sectional studies

|  | Item No | Recommendation |
| --- | --- | --- |
| **Title and abstract** | 1 | (*a*) Indicate the study’s design with a commonly used term in the title or the abstract  *Predictors of pre-marital HIV testing and intention to sero-sort marital partners; risks and consequences:* *Findings from a population-based study in Cameroon* |
|  |  | (*b*) Provide in the abstract an informative and balanced summary of what was done and what was found  *See text of the Abstract* |
| Introduction | | |
| Background/rationale | 2 | Explain the scientific background and rationale for the investigation being reported  *Introduction, paragraphs 1-2* |
| Objectives | 3 | State specific objectives, including any prespecified hypotheses  *Introduction, paragraph 6* |
| Methods | | |
| Study design | 4 | Present key elements of study design early in the paper  *Methods section, paragraph 1* |
| Setting | 5 | Describe the setting, locations, and relevant dates, including periods of recruitment, exposure, follow-up, and data collection  *Methods, paragraphs 1-3* |
| Participants | 6 | (*a*) Cross-sectional study—Give the eligibility criteria, and the sources and methods of selection of participants  *Methods-Study population & sampling, paragraph 1* |
|  |  | (*b*) For matched studies, give matching criteria and number of exposed and unexposed  *N/A* |
| Variables | 7 | Clearly define all outcomes, exposures, predictors, potential confounders, and effect modifiers. Give diagnostic criteria, if applicable  *Methods, see section on Measures: Dependent & Independent variables* |
| Data sources/ measurement | 8* | For each variable of interest, give sources of data and details of methods of assessment (measurement). Describe comparability of assessment methods if there is more than one group  *Methods, see section on Measures: Dependent & Independent variables* |
| Bias | 9 | Describe any efforts to address potential sources of bias  *Methods; Study population and sampling*  *We used a modified version of cluster sampling to select a representative sample of the study population. We minimised selection bias by inviting all respondents in selected households who met survey eligibility to participate in the study.* |
| Study size | 10 | Explain how the study size was arrived at  *Methods section: see Study population and sampling* |
| Quantitative variables | 11 | Explain how quantitative variables were handled in the analyses. If applicable, describe which groupings were chosen and why  *Methods, paragraphs 4-8* |
| Statistical methods | 12 | (*a*) Describe all statistical methods, including those used to control for confounding  *Methods section, under statistical analysis* |
|  |  | (*b*) Describe any methods used to examine subgroups and interactions  *No subgroup analysis was conducted.* |
|  |  | (*c*) Explain how missing data were addressed  *There were no known missing data.* |
|  |  | (*d*) *Cross-sectional study*—If applicable, describe analytical methods taking account of sampling strategy  *Methods section, under statistical analysis* |
|  |  | (*e*) Describe any sensitivity analyses  *NA* |
| Results | | |
| Participants | 13* | (a) Report numbers of individuals at each stage of study—eg numbers potentially eligible, examined for eligibility, confirmed eligible, included in the study, completing follow-up, and analysed  *Results, paragraph 1 (Demographic & health characteristics of respondents)* |
|  |  | (b) Give reasons for non-participation at each stage  *Methods section; under data collection ( last sentence).* |
|  |  | (c) Consider use of a flow diagram  *NA* |
| Descriptive data | 14* | (a) Give characteristics of study participants (eg demographic, clinical, social) and information on exposures and potential confounders  *Results section, paragraph 1* |
|  |  | (b) Indicate number of participants with missing data for each variable of interest  *There was no missing data.* |
|  |  | (c) Summarise follow-up time (eg, average and total amount)  *NA* |
| Outcome data | 15* | Cross-sectional study—Report numbers of outcome events or summary measures  *See Results section* |
| Main results | 16 | (*a*) Give unadjusted estimates and, if applicable, confounder-adjusted estimates and their precision (eg, 95% confidence interval). Make clear which confounders were adjusted for and why they were included  *Results, paragraph 2. No confounders were adjusted* |
|  |  | (*b*) Report category boundaries when continuous variables were categorized  *Results section, paragraph 1: The variable age was categorised as 21-25, 26-30, 31-35 years* |
|  |  | (*c*) If relevant, consider translating estimates of relative risk into absolute risk for a meaningful time period  *NA* |
| Other analyses | 17 | Report other analyses done—eg analyses of subgroups and interactions, and sensitivity analyses  *NA* |
| Discussion | | |
| Key results | 18 | Summarise key results with reference to study objectives  *Discussion, paragraphs 1* |
| Limitations | 19 | Discuss limitations of the study, taking into account sources of potential bias or imprecision. Discuss both direction and magnitude of any potential bias  *Discussion, paragraphs 10* |
| Interpretation | 20 | Give a cautious overall interpretation of results considering objectives, limitations, multiplicity of analyses, results from similar studies, and other relevant evidence  *Discussion, paragraphs 1-9* |
| Generalisability | 21 | Discuss the generalisability (external validity) of the study results  *Discussion, paragraph 10* |
| Other information | | |
| Funding | 22 | Give the source of funding and the role of the funders for the present study and, if applicable, for the original study on which the present article is based  *No funding was received for this study.* |

*Give information separately for exposed and unexposed groups.

**Note:** An Explanation and Elaboration article discusses each checklist item and gives methodological background and published examples of transparent reporting. The STROBE checklist is best used in conjunction with this article (freely available on the Web sites of PLoS Medicine at http://www.plosmedicine.org/, Annals of Internal Medicine at http://www.annals.org/, and Epidemiology at http://www.epidem.com/). Information on the STROBE Initiative is available at http://www.strobe-statement.org.
