# Supplementary material for: Predictors of willingness to accept pre-marital HIV testing and intention to sero-sort marital partners; risks and consequences: Findings from a population-based study in Cameroon
Source: PLoS One. 2018 Dec 19;13(12):e0208890. doi: 10.1371/journal.pone.0208890 (PMC6300297; doi:10.1371/journal.pone.0208890)
Supplement: S1 Questionnaire — (DOCX) [file pone.0208890.s002.docx]

**QUESTIONNAIRE**

**Awareness of HIV status, risk perceptions and attitudes towards pre-marital HIV counseling and testing among unmarried youth in two major cities in the South West Region of Cameroon**

**FOR OFFICE USE ONLY**

**Questionnaire ID:_________City Code**:________**Date :_________**

**Thank you for accepting to take part in this research study. Please answer each question by placing a tick [✓] in the box near your preferred answer. This study is anonymous and we will maintain confidentiality of any information collected. There will be no way to identify you as you should NOT write your name on the questionnaire. It will take you about 15 minutes to complete the questionnaire. Please answer the questions as truthfully as you can as that will help us answer the research questions.**

**SECTION A: AWARENESS OF HIV STATUS**

*Instructions: This section addresses prior HIV testing. We would like you to provide us some basic information about your knowledge of HIV status.*

1. **Have you ever had an HIV test? (Please tick one only)**

**2. Why have you not have an HIV test?**

❒_1_ I did not feel ready to do a test

❒_2_ I felt that I was not infected

❒_3_ I think I am not at risk of getting HIV

❒_4_ Afraid to discover a positive HIV test

❒_5_Others (Please specify)____________

**🡪(Now Skip to section B)**

❒_1_ **No**

❒_2_Yes🡪(Skip to Q3)

**3. When was the last time you had an HIV test? (Please tick one only)**

❒_1_Less than 6 months ago

❒_2_ 7 to 12 months ago

❒_3_ One year ago or more

**4. What was the reason why you did your last HIV test? (Tick all that apply)**

❒_1_For routine check-up

❒_2_Had unprotected sex and just wanted to be sure

❒_3_I was sick and was requested by the doctor

❒_4_For visa requirements

❒_5_For research purposes

❒_6_Others (Specify) __________________________

**5. Did you collect your results after doing the test?**

**6. Why did you not collect your test result?**

❒_1_I was not given my results

❒_2_ I didn’t want to know my status

❒_3_I did not have time to wait for the results

❒_4_ Others (Please specify) __________

❒_1_**No**

❒_2_**Yes🡪(Go to Section B)**

**SECTION B: HIV/AIDS RISK PERCEPTIONS**

*Instructions: This section examines your HIV/AIDS related risk perception Please tick [ ✓] only one box for each question.*

**7. Do you know of someone currently living with HIV? (Please tick one only)**

❒_1_ No

❒_2_ Yes

**8. Do you know of someone who has died of AIDS? (Please tick one only)**

❒_1_ No

❒_2_ Yes

**9. How would you rate your personal risk of contracting HIV? (Please tick one only)**

❒_1_No risk ❒_2_Low risk ❒_3_Moderate risk ❒_4_High risk

**10. How concerned are you about HIV/AIDS to each of the following items? (Please tick [✓] only one box for each item below)**

| Item | Not at all concerned | A little concerned | Very concerned | Extremely concerned |
| --- | --- | --- | --- | --- |
| **10.1 Yourself** | ❒_1_ | ❒_2_ | ❒_3_ | ❒_4_ |
| **10.2 Your family** | ❒_1_ | ❒_2_ | ❒_3_ | ❒_4_ |
| **10.3 Your community** | ❒_1_ | ❒_2_ | ❒_3_ | ❒_4_ |
| **10.4 Your country** | ❒_1_ | ❒_2_ | ❒_3_ | ❒_4_ |

**11. How serious of a threat is HIV/AIDS to each of the following items? (Please tick [✓] only one box for each item below)**

| Item | Not at all Serious | A little Serious | Very Serious | Extremely Serious |
| --- | --- | --- | --- | --- |
| **11.1 Our current generation** | ❒_1_ | ❒_2_ | ❒_3_ | ❒_4_ |
| **11.2 Future generation** | ❒_1_ | ❒_2_ | ❒_3_ | ❒_4_ |

**SECTION C: HOLISTIC AFFECT AND IMAGERY OF HIV/AIDS**

*Instructions: This section examines the good or bad feelings or the positive or negative feelings you have about HIV/AIDS. Please tick [ ✓] only one box for each question.*

**12. Do you have any negative feelings about HIV/AIDS? (Please tick one only)**

❒_1_ **No** (Skip to Q14)

**13. How would you rate the strength of your negative feelings? (Please tick one only)**

❒_1_Slightly negative

❒_2_Somewhat negative

❒_3_Very negative

❒_4_Extremely negative

❒_2_ **Yes**

**14. Do you have any positive feelings about HIV? (Please tick one only)**

❒_1_ No (Skip to Q16)

**15. How would you rate the strength of your positive feelings? (Please tick one only)**

❒_1_Slightly positive

❒_2_Somewhat positive

❒_3_Very positive

❒_4_Extremely positive

❒_2_ Yes

**16. What is the first thought or image that comes to your mind when you think of HIV/AIDS? (Please write in the box below)**

**SECTION D: ATTITUDES TOWARDS PRE-MARITAL HIV COUNSELLING AND TESTING**

***Instructions****:* This section describes what some people think about having an HIV test before marriage. Please indicate the extent to which you agree or disagree with each of the following statements by writing the appropriate response number in the column to the right of the statement. Kindly use the following response format in making your ratings for each statement:

*5 = Strongly Agree;*

*4 = Agree;*

*3 = Neither Agree Nor Disagree;*

*2 = Disagree;*

*1 = Strongly Disagree*

| *No.* | *Statement* | *Rating (Number)* |
| --- | --- | --- |
| 17. | I think HIV testing before marriage is not important in my society |  |
| 18. | I think having an HIV test before marriage is a waste of time |  |
| 19. | I think HIV testing before marriage concerns only some people |  |
| 20. | HIV testing before marriage should be encouraged in my society |  |
| 21. | HIV testing before marriage can reduce HIV transmission among partners |  |
| 22. | HIV testing before marriage can reduce the number of children born with HIV |  |
| 23. | HIV testing before marriage should only be promoted within the church community |  |
| 24. | When a boy and a girl love each other there is no need for them to do an HIV test before marriage |  |
| 25. | When a boy and girl have known each other for a long time there is no need for them to have an HIV test before marriage |  |
| 26. | HIV testing before marriage can protect future generations |  |

**SECTION E: WILLINGNESS TO HAVE AN HIV TEST BEFORE MARRIAGE AND EMOTIONAL REACTIONS**

***Instructions:*** *In this section, we would like to know about your willingness to have an HIV test before marriage and any emotional responses. Please note that all responses will be keep confidential.*

1. **Would you be willing to have an HIV test before marriage, if requested?**

**28. Why would you refuse to an HIV test before marriage? (Tick all that apply)**

❒_1_Fear of stigma

❒_2_Fear of discrimination

❒_3_Concern about confidentiality

❒_4_Costs associated with the test

❒_5_Other (Specify) _____________

❑_1_No

❑_2_Yes

**29. “How would you feel if a pre-marital HIV test shows that you are HIV negative while your partner is HIV positive?” (Please tick as many below as you will feel)**

❒_1_Disappointed

❒_2_ Sad

❒_3_Anxious

❒_4_Depressed

❒_5_Worry

❒_6_ Afraid

❒_7_Angry

_8_ Feel Normal

❒_9_Others (Specify) _______

**30. If the results of a pre-marital HIV test show that you are HIV negative while your partner is HIV positive, would you still marry your HIV positive partner? (Please tick one only)**

❒_1_ No (Skip to Q32)

**31. Why would you still accept to marry this person?**

_1_Because I love the person

_2_Because there is treatment now for HIV

_3_ Others (Specify) _________________

❒_2_ Yes

**32. Would you seek support to assist you manage this situation? (Please tick one only)**

**❒**_1_No (Skip to **Section F**)

**33. Where will you seek support? (Tick all that apply)**

❒_1_Friend

❒_2_Family member

❒_3_Counsellor

❒_4_Medical doctor

❒_5_Religious leader

❒_6_Others (Specify) _______________

**❒**_2_Yes

**SECTION F: SOCIO-DEMOGRAPHIC CHARACTERISTICS**

*Instructions: In this section, we would like you to provide some basic information about yourself. Please remember that all responses will be treated as confidential.*

**34. What is your current age?** _________**(years)**

1. **Gender (Please tick one only)**

❑_1_ Female

❑_2_ Male

1. **Which of the following best describes your level of education? (Please tick one only)**

❒_1_Completed primary school

❒_2_Completed secondary school (GCE O/L)

❒_3_Completed high school (GCE A/L)

❒_4_Advanced diploma (HND, etc.)

❒_5_Bachelor’s degree and above

1. **Which of the following best describes your current employment status? (Please tick one only)**

❑_1_Student

❒_2_Unemployed

❒_3_Employed full-time

❒_4_Employed part-time

❒_5_ Self-employed

1. **What is your current relationship status? (Please tick one only)**

❑_1_I am not in a relationship (Skip to Q40)

**39. Do you know the HIV status of your current partner? (Please tick one only)**

❒_1_No

❒_2_Yes

❑_2_ I am in a relationship

**40. What is your current religious denomination? (Please tick one only)**

❒_1_Roman Catholic

❒_2_Presbyterian

❒_3_Pentecostal/Born Again

❒_4_Baptist

❒_5_Apostolic

❒_6_Islam/Moslem

❒_7_Others (Please specify) ___________________

**STOP HERE**

**On behalf of the research team, we thank you very much for taking part in this study.**
