# Supplementary material for: Predictors of willingness to accept pre-marital HIV testing and intention to sero-sort marital partners; risks and consequences: Findings from a population-based study in Cameroon
Source: PLoS One. 2018 Dec 19;13(12):e0208890. doi: 10.1371/journal.pone.0208890 (PMC6300297; doi:10.1371/journal.pone.0208890)
Supplement: S1 Table — (DOCX) [file pone.0208890.s004.docx]

**S1 Table. Univariate logistic regression analysis of factors associated with willingness to accept pre-marital HIV testing**

| **Independent variables** | **Kumba** | | **Buea** | |
| --- | --- | --- | --- | --- |
|  | **OR (95% CI)** | **P-value** | **OR (95% CI)** | **P-value** |
| **Age group (yrs)** |  |  |  |  |
| 21-25 | 1.0 |  | 1.0 |  |
| 26-30 | 0.66 (0.34-1.29) | 0.230 | 1.00 (0.43-2.36) | 0.991 |
| 31-35 | 0.46 (0.21-0.97) | 0.041 | 0.41 (0.18-0.93) | 0.032 |
| **Gender** |  |  |  |  |
| Female | 1.0 |  | 1.0 |  |
| Male | 0.41 (0.21-0.81) | 0.010 | 0.45 (0.21-0.94) | 0.035 |
| **Educational attainment** |  |  |  |  |
| Primary school | 1.0 |  | 1.0 |  |
| Secondary school | 1.69 (0.71-4.04) | 1.190 | 0.56 (0.19-1.62) | 0.285 |
| High school | 1.08 (0.53-2.19) | 0.210 | 0.59 (0.23-1.55) | 0.288 |
| University | 1.03 (0.43-2.49) | 0.080 | 2.06 (0.44-9.58) | 0.352 |
| **Employment status** |  |  |  |  |
| Student | 1.0 |  | 1.0 |  |
| Unemployed | 0.26 (0.08-0.91) | 0.034 | 1.28 (0.35-4.74) | 0.703 |
| Employed^1^ | 0.35 (0.11-1.05) | 0.062 | 0.76 (0.32-1.79) | 0.532 |
| **Religion** |  |  |  |  |
| Catholic | 1.0 |  | 1.0 |  |
| Presbyterian | 0.96 (0.39-2.36) | 0.931 | 1.32 (0.49-3.51) | 0.577 |
| Pentecostal | 0.94 (0.38-2.32) | 0.905 | 2.07 (0.68-6.27) | 0.196 |
| Others^2^ | 0.39 (0.17-0.89) | 0.025 | 0.76 (0.32-1.80) | 0.540 |
| **Currently in a sexual relationship** |  |  |  |  |
| No | 1.0 |  | 1.0 |  |
| Yes | 0.88 (0.47-1.67) | 0.701 | 1.15(0.55-2.43) | 0.704 |
| **Know current sexual partner’s HIV status^3^** |  |  |  |  |
| No | 1.0 |  | 1.0 |  |
| Yes | 1.51 (0.77-3.00) | 0.228 | 2.36 (1.02-5.46) | 0.045 |
| **Know someone living with HIV** |  |  |  |  |
| No | 1.0 |  | 1.0 |  |
| Yes | 0.77 (0.41-1.45) | 0.424 | 0.91 (0.45-2.17) | 0.783 |
| **Know someone who has died of AIDS** |  |  |  |  |
| No | 1.0 |  | 1.0 |  |
| Yes | 0.96 (0.53-1.71) | 0.883 | 1.09 (0.55-2.17) | 0.801 |
| **Self-perceived risk of contracting HIV** |  |  |  |  |
| No risk | 1.0 |  | 1.0 |  |
| Small risk | 1.82 (0.80-4.14) | 0.151 | 1.88 (0.67-5.24) | 0.674 |
| Moderate risk | 1.16 (0.53-2.54) | 0.704 | 0.48 (0.21-1.16) | 0.206 |
| High risk | 1.12 (0.50-2.49) | 0.781 | 1.95 (0.45-8.52) | 0.447 |
| **Previously tested for HIV** |  |  |  |  |
| No | 1.0 |  | 1.0 |  |
| Yes | 6.7(3.54-12.67) | <0.001 | 3.06 (1.52-6.17) | 0.002 |

Notes:

^1^Employed: Part-time, Full time or self-employed; ^2^Other Religion included: Baptist, Islam, Apostolic, Jehovah’s Witness etc ^3^ Only for those who were currently in a sexual relationship
