# Supplementary material for: Predictors of willingness to accept pre-marital HIV testing and intention to sero-sort marital partners; risks and consequences: Findings from a population-based study in Cameroon
Source: PLoS One. 2018 Dec 19;13(12):e0208890. doi: 10.1371/journal.pone.0208890 (PMC6300297; doi:10.1371/journal.pone.0208890)
Supplement: S2 Table — (DOCX) [file pone.0208890.s005.docx]

**S2 Table. Univariate logistic regression analysis of factors associated with acceptance to marry an HIV positive partner**

| **Independent variables** | **Kumba** | | **Buea** | |
| --- | --- | --- | --- | --- |
|  | **OR (95% CI)** | **P-value** | **OR (95% CI)** | **P-value** |
| **Age group (yrs)** |  |  |  |  |
| 21-25 | 1.0 |  | 1.0 |  |
| 26-30 | 0.91(0.65-1.27) | 0.584 | 0.98 (0.68-1.41) | 0.934 |
| 31-35 | 1.41 (0.94-2.10) | 0.097 | 1.11 (0.72-1.71) | 0.644 |
| **Gender** |  |  |  |  |
| Female | 1.0 |  | 1.0 |  |
| Male | 0.81 (0.60-1.09) | 0.180 | 0.67 (0.49-0.95) | 0.017 |
| **Educational attainment** |  |  |  |  |
| Primary school | 1.0 |  | 1.0 |  |
| Secondary school | 1.08 (0.72-1.64) | 0.697 | 1.28 (0.79-2.07) | 0.301 |
| High school | 0.98 (0.66-1.44) | 0.919 | 0.83 (0.54-1.29) | 0.417 |
| University | 1.04 (0.65-1.67) | 0.871 | 1.29 (0.79-2.12) | 0.304 |
| **Employment status** |  |  |  |  |
| Student | 1.0 |  | 1.0 |  |
| Unemployed | 1.25 (0.75-2.09) | 0.385 | 0.82 (0.47-1.40) | 0.478 |
| Employed^1^ | 1.35 (0.89-2.04) | 0.147 | 0.91 (0.62-1.33) | 0.630 |
| **Religion** |  |  |  |  |
| Catholic | 1.0 |  | 1.0 |  |
| Presbyterian | 1.15 (0.75-1.77) | 0.508 | 0.72 (0.46-1.12) | 0.145 |
| Pentecostal | 1.61 (1.06-2.43) | 0.025 | 0.71 (0.46-1.10) | 0.130 |
| Others^2^ | 1.21 (0.77-1.90) | 0.402 | 0.70 (0.45-1.09) | 0.119 |
| **Currently in a sexual relationship** |  |  |  |  |
| No | 1.0 |  | 1.0 |  |
| Yes | 1.02 (0.74-1.41) | 0.896 | 1.17 (0.82-1.68) | 0.379 |
| **Know current sexual partner’s HIV status^3^** |  |  |  |  |
| No | 1.0 |  | 1.0 |  |
| Yes | 1.35 (0.93-1.96) | 0.110 | 0.81 (0.53-1.22) | 0.323 |
| **Know someone living with HIV** |  |  |  |  |
| No | 1.0 |  | 1.0 |  |
| Yes | 1.24 (0.89-1.73) | 0.201 | 1.01 (0.73-1.40) | 0.944 |
| **Know someone who has died of AIDS** |  |  |  |  |
| No | 1.0 |  | 1.0 |  |
| Yes | 1.09 (0.81-1.48) | 0.533 | 1.14 (0.83-1.57) | 0.411 |
| **Self-perceived risk of contracting HIV** |  |  |  |  |
| No risk | 1.0 |  | 1.0 |  |
| Small risk | 0.75 (0.50-1.12) | 0.158 | 0.70 (0.46-1.08) | 0.109 |
| Moderate risk | 0.83 (0.54-1.26) | 0.390 | 1.67 (1.07-2.58) | 0.023 |
| High risk | 0.73 (0.47-1.14) | 0.172 | 0.86 (0.49-1.51) | 0.599 |
| **Previously tested for HIV** |  |  |  |  |
| No | 1.0 |  | 1.0 |  |
| Yes | 1.13 (0.81-1.57) | 0.462 | 1.17 (0.83-1.66) | 0.360 |

Notes:

^1^Employed: Part-time, Full time or self-employed; ^2^Other Religion included: Baptist, Islam, Apostolic, Jehovah’s Witness etc ^3^ Only for those who were currently in a sexual relationship
